# Long-term outcomes of autoimmune pancreatitis: a multicentre, international analysis

**DOI:** 10.1136/gutjnl-2012-303617

**Published:** 2012-12-11

**Authors:** Phil A Hart, Terumi Kamisawa, William R Brugge, Jae Bock Chung, Emma L Culver, László Czakó, Luca Frulloni, Vay Liang W Go, Thomas M Gress, Myung-Hwan Kim, Shigeyuki Kawa, Kyu Taek Lee, Markus M Lerch, Wei-Chih Liao, Matthias Löhr, Kazuichi Okazaki, Ji Kon Ryu, Nicolas Schleinitz, Kyoko Shimizu, Tooru Shimosegawa, Roy Soetikno, George Webster, Dhiraj Yadav, Yoh Zen, Suresh T Chari

**Affiliations:** 1 Division of Gastroenterology and Hepatology, Mayo Clinic, Rochester, Minnesota, USA; 2 Department of Internal Medicine, Tokyo Metropolitan Komagome Hospital, Tokyo, Japan; 3 Department of Internal Medicine, GI Unit, Massachusetts General Hospital, Boston, Massachusetts, USA; 4 Department of Internal Medicine, Institute of Gastroenterology, Severance Hospital, Yonsei University College of Medicine, Seoul, Korea; 5 Translational Gastroenterology Unit, John Radcliffe Hospital, Oxford, UK; 6 First Department of Internal Medicine, University of Szeged, Szeged, Hungary; 7 Department of Medicine, Biomedical and Surgical Sciences, University of Verona, Verona, Italy; 8 Center for Excellence in Pancreatic Disease, David Geffen School of Medicine at University of California, Los Angeles, California, USA; 9 Department of Gastroenterology, Endocrinology, Metabolism and Infectiology, Philipps University of Marburg, Marburg, Germany; 10 Department of Internal Medicine, Asan Medical Center, University of Ulsan College of Medicine, Seoul, Korea; 11 Center for Health, Safety and Environmental Management, Shinshu University, Matsumoto, Japan; 12 Department of Medicine, Samsung Medical Center, Sungkyunkwan University School of Medicine, Seoul, Korea; 13 Department of Medicine A, University Medicine Greifswald, Greifswald, Germany; 14 Department of Internal Medicine, National Taiwan University Hospital, Taipei, Taiwan; 15 Department of Surgical Gastroenterology, Karolinska Institutet & Karolinska University Hospital, Stockholm, Sweden; 16 Department of Gastroenterology and Hepatology, Kansai Medical University, Osaka, Japan; 17 Department of Internal Medicine, Seoul National University College of Medicine, Seoul, Korea; 18 Department of Internal Medicine, Aix-Marseille University, Marseille, France; 19 Department of Gastroenterology, Tokyo Women's Medical University, School of Medicine, Tokyo, Japan; 20 Division of Gastroenterology, Tohoku University Graduate School of Medicine, Sendai, Japan; 21 Department of Internal Medicine, Affiliated Stanford University, Palo Alto, California, USA; 22 Department of Gastroenterology, University College Hospital, London, UK; 23 Division of Gastroenterology, Hepatology and Nutrition, University of Pittsburgh, Pennsylvania, USA; 24 Institute of Liver Studies, King's College Hospital, London, UK

**Keywords:** Autoimmune Disease, Pancreatic Cancer, Pancreato-Biliary Disorders

## Abstract

**Objective:**

Autoimmune pancreatitis (AIP) is a treatable form of chronic pancreatitis that has been increasingly recognised over the last decade. We set out to better understand the current burden of AIP at several academic institutions diagnosed using the International Consensus Diagnostic Criteria, and to describe long-term outcomes, including organs involved, treatments, relapse frequency and long-term sequelae.

**Design:**

23 institutions from 10 different countries participated in this multinational analysis. A total of 1064 patients meeting the International Consensus Diagnostic Criteria for type 1 (n=978) or type 2 (n=86) AIP were included. Data regarding treatments, relapses and sequelae were obtained.

**Results:**

The majority of patients with type 1 (99%) and type 2 (92%) AIP who were treated with steroids went into clinical remission. Most patients with jaundice required biliary stent placement (71% of type 1 and 77% of type 2 AIP). Relapses were more common in patients with type 1 (31%) versus type 2 AIP (9%, p<0.001), especially those with IgG4-related sclerosing cholangitis (56% vs 26%, p<0.001). Relapses typically occurred in the pancreas or biliary tree. Retreatment with steroids remained effective at inducing remission with or without alternative treatment, such as azathioprine. Pancreatic duct stones and cancer were uncommon sequelae in type 1 AIP and did not occur in type 2 AIP during the study period.

**Conclusions:**

AIP is a global disease which uniformly displays a high response to steroid treatment and tendency to relapse in the pancreas and biliary tree. Potential long-term sequelae include pancreatic duct stones and malignancy, however they were uncommon during the study period and require additional follow-up. Additional studies investigating prevention and treatment of disease relapses are needed.

**Significance of this study**

- What is already known on this subject?
- Autoimmune pancreatitis (AIP) is a treatable form of chronic pancreatitis that is felt to be responsive to steroid treatment.
- There are few long-term data regarding response to treatment and subsequent disease sequelae.

- What are the new findings?
- Disease relapses are common after steroid discontinuation, and typically occur in the pancreas and/or biliary tract.
- Pancreatic duct stones are relatively uncommon, but are seen more frequently in patients with at least one disease relapse.
- The occurrence of incident cancers following AIP diagnosis appears to be uncommon.

- How might it impact on clinical practice in the foreseeable future?
- Since disease relapses are common, additional studies are needed to compare different treatment strategies for maintaining disease remission.
- Further investigations are needed to understand if the risk of cancer is increased compared with the general population.

## Introduction

Autoimmune pancreatitis (AIP) is a unique form of chronic pancreatitis with characteristic histological features, frequent elevations of serum IgG4 levels and a predictable response to steroid therapy. Although the identification of a steroid-responsive form of chronic pancreatitis was initially reported in 1995 by Yoshida *et al*, there was minimal progress in understanding this disease until a serum biomarker (IgG4 antibody) was identified by Hamano *et al*.^1^ ^2^ Over the last decade significant progress has been made in understanding this disease, including identification of two distinct histological subtypes, with different clinical phenotypes (termed type 1 and type 2 AIP), incorporation of seemingly unrelated diseases within the spectrum of IgG4-related disease (of which AIP is the pancreas manifestation) and treatment of refractory patients with rituximab.^3–6^

Despite these advances, many questions remain unanswered. Although patients respond initially to steroid therapy, many patients will develop disease relapse either during steroid taper or following steroid discontinuation. Reported rates of disease relapse have ranged from 15–60% in various series.^4^ ^7–10^ Although there is general agreement that steroids are the ideal initial treatment, there is no clear consensus regarding treatment for disease relapses.

Due to the recent recognition of patients with this condition, the long-term sequelae of the disease are largely unknown. Follow-up data are recently becoming available, permitting the present analysis. In an effort to better understand these knowledge gaps we set out to perform an international analysis of patients with type 1 and type 2 AIP. One previous study evaluated the distribution of AIP subtypes worldwide, however multiple diagnostic criteria were used based on the country of origin.^11^ Recently a multinational group met and agreed upon diagnostic criteria, termed International Consensus Diagnostic Criteria (ICDC).^12^ This classification scheme categorises diagnostic evidence into one of two levels of confidence in the following categories: pancreatic parenchymal imaging, imaging of the pancreatic duct (ie, endoscopic retrograde pancreatogram), serum IgG4 level, other organ involvement of IgG4-related disease, histology of the pancreas (from core biopsy or resection) and response to steroid treatment. We specifically set out to gain additional understanding of the current burden of AIP at several large, academic institutions using the ICDC, and to describe the long-term outcomes of this disease including organs involved, treatments, relapse frequency and long-term sequelae.

## Methods

A total of 31 institutions were invited to participate in this study on the basis of their scientific merit in this field, or established experience in management of AIP; ultimately 23 institutions from 10 different countries participated. The Tokyo Metropolitan Komagome Hospital in Japan and Mayo Clinic in the USA served as the coordinating centres. The study was approved by the institutional review board of Tokyo Metropolitan Komagome Hospital and was in compliance with the Declaration of Helsinki.

In centres with existing patient databases, patient follow-up data were updated and retrieved. If data were not available, investigators retrospectively reviewed paper and/or electronic medical records or contacted patients by telephone for data collection. Each centre independently reviewed histological, radiographic, and clinical records of subjects with suspected AIP. Subjects classified as either definite or probable type 1 or type 2 AIP according to the ICDC were selected for this study (see online supplementary tables S1–S4).^12^ The two subtypes are definitively distinguished based on their histology in which type 1 AIP (also known as lymphoplasmacytic sclerosing pancreatitis) demonstrates lymphoplasmacytic infiltration, obliterative phlebitis, storiform fibrosis and abundant IgG4-postive cells, while type 2 AIP (also known as GEL+ pancreatitis or idiopathic duct-centric chronic pancreatitis) shows granulocytic infiltration of the duct wall (termed GEL) and absent or minimal IgG4-postive cells. Additionally, type 2 AIP patients are unlikely to have serum IgG4 elevation or other organ involvement. Site data through 1 January 2012 were compiled using a standardised data collection form, then submitted to the lead investigator (TK) for analysis.

### Definitions

For the purposes of this study, proximal biliary was defined as involvement of either intrahepatic bile ducts or the extrahepatic common bile duct proximal to the head of the pancreas. When it occurred in the context of type 1 AIP it was referred to as IgG4-related sclerosing cholangitis (IgG4-related SC)). On the other hand, distal biliary disease referred to disease isolated to the intrapancreatic portion of the common bile duct. Serum IgG4 values vary depending on the assay used, so normal levels were recognised as those less than the upper limit of normal for the lab where the test was performed. Pancreatic duct stones were identified with the use of either cross-sectional imaging or endoscopic retrograde pancreatography.

### Treatment regimens

A wide variety of steroid regimens were employed for induction and maintenance of remission. For the initial dose of steroids, all centres used either a weight-based strategy (0.6 mg/kg/day of prednisolone) or fixed-dose regimen (30–40 mg/day) that were roughly equivalent for treatment of a 70 kg individual. Tapering strategies ranged from 5–10 mg decrease every 1–2 weeks. All Asian centres (n=10) used a maintenance strategy of low-dose (2.5–5 mg/day) prednisolone, which was continued for anywhere from 6 months to 3 years. In general, the European and North American groups tapered the steroids off within 3 months and did not provide maintenance doses of steroids. Multiple centres elected to use immunomodulator drugs instead of low-dose steroids for maintenance therapy (n=5). In the four centres treating more than five subjects with this strategy, azathioprine (2 mg/kg/day) was the preferred agent and was used for a variable duration of time (1–3 years).

Many patients initially underwent surgery either due to the absence of typical features of AIP or clinical presentation prior to the recognition of AIP as a disease entity. Surgeries were performed for resection of mass-forming lesions (ie, pancreaticoduodenectomy or distal splenectomy) or palliative bypass (ie, gastrojejunostomy) for those with an apparently unresectable cancer. Surgery was not intentionally performed as primary treatment for AIP. A number of patients were treated conservatively, without the use of steroids or surgery. Supportive care was provided for a variety of reasons including asymptomatic disease, severe comorbid disease (eg, metastatic cancer) or patient preference.

### Statistical analyses

Continuous variables were compared using Student t test, χ^2^ test and Fisher's exact t test (when one or more expected cell frequencies were <5) were used for comparison of proportions. A p value <0.05 was considered statistically significant.

## Results

### AIP subject characteristics

**Figure 1:**
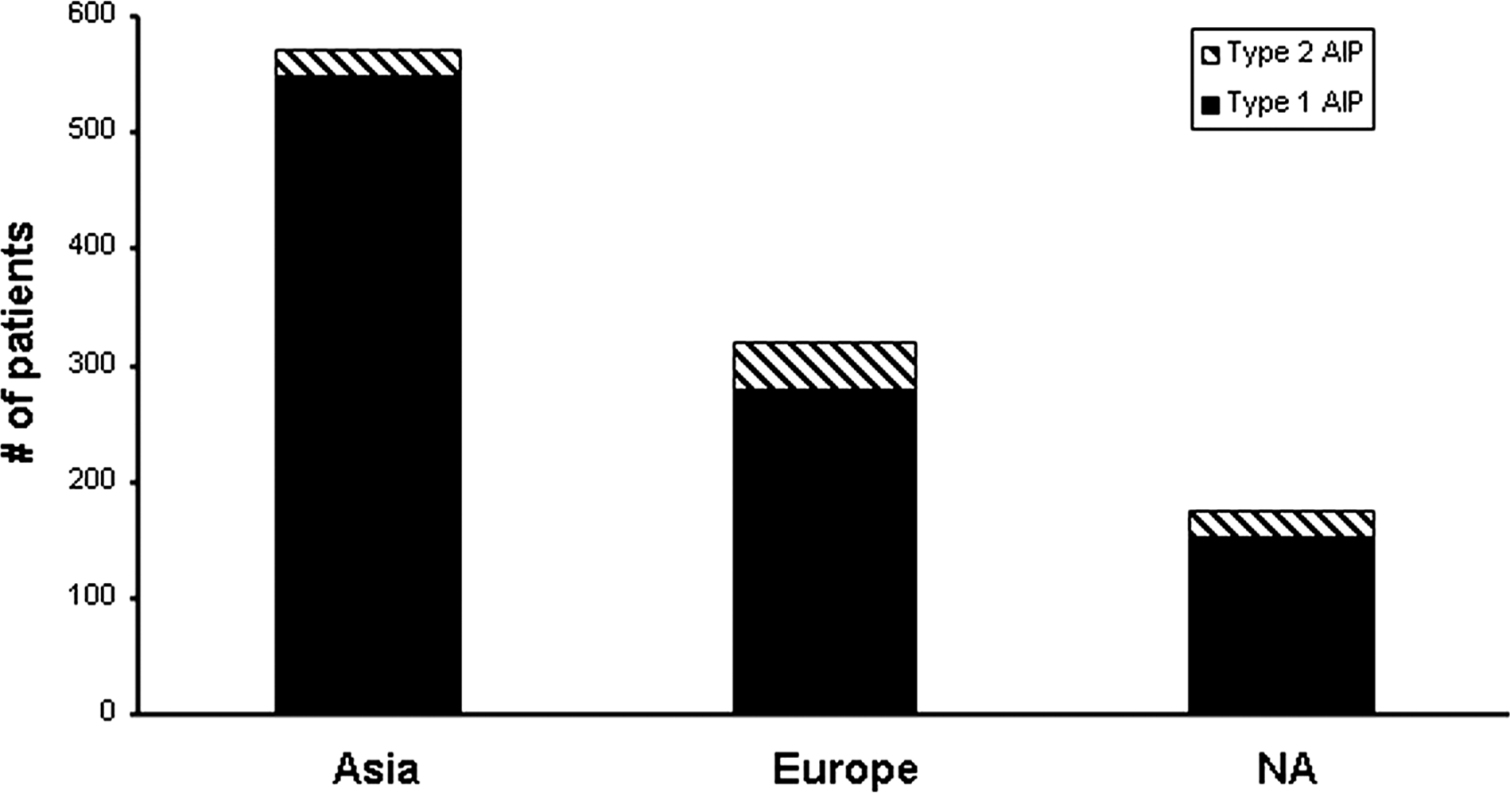
Regional distribution of type 1 and type 2 autoimmune pancreatitis based on the country of diagnosis. NA, North America.

A total of 1064 subjects were identified, 978 with type 1 AIP and 86 with type 2 AIP. The average age of subjects at diagnosis was 61.4 and 39.9 years for type 1 and type 2 subjects, respectively. The proportion of males was 77% in type 1 subjects and 55% in type 2 subjects (p<0.001). The proportion of patients diagnosed with type 2 AIP was lower in Asian countries (3.7%) compared with European (12.9%, p<0.001) and North American (13.7%, p<0.001) countries (figure 1).

### Treatment response

The majority (74%) of subjects with type 1 AIP were initially treated with steroids, rather than surgical or conservative treatments, in comparison with type 2 subjects in which only 62% were treated with steroids (p=0.01). Remission was successfully induced in almost all subjects with type 1 and type 2 AIP (table 1). The per cent of subjects achieving remission was higher in type 1 subjects who received intervention (either steroids or surgery) (99.2%) compared with those who were managed conservatively (55.2%, p<0.001). However, initial remission rates were similar in patients with type 2 AIP who received intervention compared with conservative management (83.5% vs 66.7%, respectively, p=0.29). Interestingly many of the patients who underwent palliative surgical bypass achieved successful clinical remission; however the total number of cases was small. Initial treatment strategies and indications for treatment and concurrent therapies used in those receiving steroid treatment are also shown in table 1. Treatment for diabetes mellitus was given to a minority of patients prior to steroid treatment. However, biliary stenting was performed for most subjects presenting with jaundice. In subjects with type 1 AIP, jaundice (63%) was the most common indication, followed by abdominal pain with or without biochemical pancreatitis. In those with type 2 AIP, abdominal pain and inflammatory bowel disease were major indications.

**Table 1:** Initial treatment strategies and treatment details for those treated with steroids

	Type 1 AIP (n=901†)		Type 2 AIP (n=85†)		
	Successful remission, n	%	Successful remission, n	%	
Initial treatment					
Steroids	681/684	99.6	48/52	92.3	
Surgical resection	125/127	98.4	17/25	68.0	
Palliative surgical bypass	22/23	95.7	1/2	50.0	
Conservative	37/67	55.2	4/6	66.7	

	**Type 1 AIP (n=724)**		**Type 2 AIP (n=53)**		
	**n**	**%**	**n**	**%**	p Value*

Indications for steroid treatment					
Jaundice	458	63	13	25	<0.001
Pancreatitis/abdominal pain	198	27	34	64	<0.001
Abnormal imaging (diffuse pancreatic enlargement, pancreas mass)	71	10	0	–	0.01
Salivary gland enlargement	49	7	0	–	0.04
Diagnostic steroid trial	46	6	4	8	0.77
Retroperitoneal fibrosis	17	2	0	–	0.62
IgG4-related renal disease	9	1.2	0	–	0.99
Lymphadenopathy	6	0.8	0	–	0.99
IgG4-related lung disease	4	0.6	0	–	0.99
Inflammatory bowel disease	1	0.1	23	48	<0.001
Other (hyperglycaemia, weight loss, etc)	20	3	0	–	
Diabetes management					
Oral medications	99/596	17	6/46	13	0.53
Insulin therapy	136/596	23	4/46	9	0.03
Endoscopic management (for subjects with jaundice)					
Biliary stent placement	351/492	71	10/13	77	0.77

*p Values represent comparison of proportions between patients with type 1 and type 2 AIP using χ^2^ and Fisher's exact t test, when appropriate.

†Seventy-seven subjects with type 1 AIP and one subject with type 2 AIP are not displayed in the table due to pending response to treatment at study closure.

AIP, Autoimmune pancreatitis.

In subjects with type 1 AIP and abnormal serum IgG4 levels (n=446) prior to steroids, the serum level decreased in 427 (95.7%) subjects and returned to within normal limits for 204 (45.7%). Of 609 type 1 AIP subjects with pancreatic enlargement at the time of diagnosis, the parenchyma appeared normal in 400 (65.7%), atrophic in 173 (28.4%) and persistently enlarged in 35 (5.9%) subjects following steroid treatment. In contrast, 50 type 2 AIP subjects had pancreatic enlargement at diagnosis. Following treatment the appearance returned to normal in the majority (43/50, 86%) with progression to atrophy in the remaining seven subjects.

### Relapse data

Of the 978 subjects with type 1 disease, a total of 302 (31%) subjects experienced at least one disease relapse during the study period, compared with 8 (9%, p<0.001) subjects with type 2 AIP (table 2). The vast majority of relapse episodes occurred in steroid treated subjects following steroid discontinuation (67%), as compared with during the steroid taper (15%) or while on maintenance steroids (18%). Most relapses occurred in the biliary system or pancreas for type 1 AIP, while relapses in type 2 AIP were limited to the pancreas.

**Table 2:** Distribution of disease relapse episodes according to initial treatment strategies, and location and frequency for those treated with steroids

	Type 1 AIP		Type 2 AIP	
	Relapse, n	%	Relapse, n	%
Initial treatment				
Steroids	245/684	35.8	8/52	15.3
Surgical resection	35/116	30.2	0/25	0
Palliative surgical bypass	11/23	47.8	0/2	0
Conservative	11/57	19.3	0/6	0
*Disease relapses following steroid treatment*				
Location of relapse	n=245 episodes		n=8 episodes	
Biliary system	124	50.6	–	–
Pancreas	107	42.9	8	100
Salivary	18	7.3	–	–
Lung	11	4.5	–	–
Lymphadenopathy	4	1.6	–	–
Renal	3	1.2	–	–
Other (RPF or NOS)	13	5.3	–	–
Frequency per subject				
One relapse	189	77.1	8	100
Two relapses	39	15.9	–	–
Three relapses	13	5.3	–	–
≥4 relapses	4	1.6	–	–

AIP, Autoimmune pancreatitis; RPF, retroperitoneal fibrosis; NOS, not otherwise specified.

### Predictors of relapse

The proportion of subjects having a relapse was similar in those with persistently abnormal IgG4 levels following steroids compared with those with a normal level (32.7% vs 31.4%, respectively, p=0.77). Likewise, the proportion of subjects with at least one relapse was similar regardless of whether they initially had diffuse (42/440, 32.3%) or focal pancreatic parenchymal enlargement (92/285, 32.3%, p=0.99). In contrast, 96/171 (56.1%) subjects with IgG4-related SC had at least one relapse, while only 142/551 (25.7%) subjects without IgG4-related SC had a relapse (p<0.001). The rates of relapse were similar in those with and without distal biliary disease (33.9% vs 31.1%, respectively, p=0.44). Since there were very few relapse episodes in subjects with type 2 AIP, a meaningful comparison of risk factors for relapse could not be completed.

### Treatment for disease relapse

Steroids were the most commonly used treatment for managing disease relapse in type 1 AIP, and inducing remission was successful in 201/210 (95%) of subjects. The addition of azathioprine was used for 68 subjects with successful induction in 56 (85%). Medications used in other subjects (n=18) included mycophenolate mofetil (n=8), cyclosporine (n=3), methotrexate, 6-mercaptopurine, cyclophosphamide and rituximab. Successful remission was achieved in 12 (86%) of these subjects with follow-up.

### Long-term sequelae in type 1 AIP

Pancreatic duct stones were uncommonly seen occurring in 46/659 (7%) subjects with follow-up imaging permitting evaluation for stone disease. Pancreatic duct stones were more likely to occur in subjects with at least one relapse, compared with those who had never had a relapse (14.4% vs 4.0%, respectively, p<0.001).

The most frequently occurring cancers during follow-up were gastric, lung and prostate (table 3). Importantly, pancreatic cancer was diagnosed in five male patients at a median age of 77 years (range 65–80) at the time of cancer diagnosis. All cancers were diagnosed more than 3 years following AIP diagnosis with the exception of one patient. His cancer was diagnosed 9 months following AIP diagnosis, which was made on the basis of diffuse pancreatic enlargement and elevation of serum IgG4 more than twice the upper limit of normal (definitive histology for type 1 AIP was confirmed on the resected pancreatic specimen). In the two patients with serum IgG4 levels obtained at the time of cancer diagnosis, it was mildly (1–2×upper limits) elevated. Eight (73%) of the subjects with gastric cancers were from study sites located in Japan or Korea, and risk factors for gastric cancer were not reported. No subjects with type 2 AIP developed an incident cancer or pancreatic duct stone during the study period.

**Table 3:** Cumulative frequency of malignancies in type 1 AIP subjects

Cancer type	Subjects, n
Gastric	11
Lung	9
Prostate	7
Colon	5
Pancreatic	5
Oesophageal	4
Cholangiocarcinoma	3
Leukaemia	3
Ovarian	2
Renal	2
Other*	6

*Other cancers with only one reported case include: testicular, gastrointestinal stromal tumour, breast, bladder, hepatocellular and adenocarcinoma of unknown primary.

AIP, Autoimmune pancreatitis.

## Discussion

This study represents the largest, multinational analysis of patients with type 1 and type 2 AIP diagnosed according to ICDC and provides insights into treatment strategies and potential long-term sequelae. Previously noted differences in clinical profiles of type 1 and type 2 AIP, including age and gender differences, were confirmed in this study.^4^ ^11^ Type 2 AIP represented a smaller proportion of AIP in Asian countries compared with European and North American countries.

Types 1 and 2 were highly-responsive to steroid treatment; however disease relapses were common in type 1, especially in those with proximal biliary disease (ie, IgG4-related SC). Most patients who required steroid therapy had predominantly pancreatobiliary disease manifestations (jaundice, abdominal pain or abnormal pancreas imaging). Although most subjects with jaundice required biliary intervention prior to steroid therapy, the need for diabetes treatment was unexpectedly low. The remission rate of treating patients following disease relapse remained high. Pancreatic duct stones were relatively uncommon, but occurred more frequently in patients with at least one disease relapse. A number of cancers occurred and further studies are needed to understand whether this represents a true increased risk for malignancy in subjects with AIP or is due to older age of type 1 patients and ascertainment bias as patients with AIP have extensive diagnostic studies and close follow-up.

Our present compilation of more than 1000 patients is the largest to date, and the number of institutions required to reach this enrolment illustrates the rarity of this disease. Since the landmark discovery that elevated serum IgG4 levels are associated with AIP the number of newly diagnosed cases of AIP has increased dramatically.^2^ The disease spectrum of IgG4-related disease, which encompasses AIP and IgG4-related SC, continues to expand also contributing additional diagnoses. It is more likely that the increasing recognition of the diseases is due to its increased awareness rather than true increase in incidence of the disease.

Since its initial description by Yoshida *et al*, type 1 AIP has been recognised as a steroid-responsive disease.^1^ The current study shows that both types of disease are characterised by very high remission rates with steroid therapy, suggesting that the diagnosis must be reconsidered in those who do not respond to steroids. Although a noteworthy proportion (55%) of patients initially managed conservatively had spontaneous disease remission, this rate was inferior to that in patients who were treated with steroids or surgery (99% remission rate). Since inflammatory pancreatic and biliary disease can progress to irreversible pancreatic insufficiency and secondary biliary cirrhosis, we feel early treatment is advisable, even in the absence of rigorous evidence-based medicine demonstrating that steroid treatment alters the natural history of AIP. In the absence of a validated induction regimen variation in steroid dosing is inevitable, but despite this remission rates were universally high across all centres. Most patients required treatment for jaundice, abdominal pain or abnormal pancreatic imaging. Interestingly, although most patients with jaundice required endoscopic biliary stenting prior to steroids, less than half of patients required treatment for diabetes. Smaller series have shown an interesting, paradoxical improvement in glycaemic control after steroid therapy, presumably due to recovered pancreatic endocrine function with treatment.^13^ ^14^ This finding, experienced anecdotally by many of the authors, led to the withholding of diabetes treatment for some patients with hyperglycaemia. Nonetheless, it is important to monitor blood sugars during steroid treatment to recognise and prevent hyperglycaemia-related morbidity.

Disease relapses in type 1 and type 2 AIP predominantly involve the pancreas and/or biliary system. Cumulative relapse rates could not be accurately calculated since time to event (ie, relapse) data were not available for most patients. However, the relapse rate in this study of 31% falls within the range (15–60%) of that in previous reports. Unfortunately due to the nature of this study, it is not statistically valid to compare relapse rates on the basis of treatment strategies used (eg, with or without maintenance steroids). Due to challenges with study enrolment, a prospective treatment trial to clarify this choice is not expected soon; so the decision must be made on the basis of the provider's familiarity with the treatment strategy, considering the side effect profile, and patients’ personal relapse histories and preferences.

For most patients in this study relapses occurred after steroid discontinuation. Patients treated again with steroids continued to respond favourably with a high remission rate. Some patients with relapses were treated with an immunomodulator (most commonly azathioprine or mycophenolate mofetil). These steroid-sparing approaches are attractive to some due to the possibility of avoiding complications from long-term steroid exposure.^6^ ^8^ ^14–16^ However, to date no large series have demonstrated either treatment effectiveness or decreased incidence of treatment-related side effects.

Two sequelae identified in other forms of chronic pancreatitis are pancreatic duct stones and pancreatic cancer. So we specifically examined the rates of these complications in this study cohort.^17^ ^18^ The occurrence of pancreatic duct stones in this study is low with higher prevalence in those with at least one relapse. Additionally, we report the first systematic collection of malignancies in patients with AIP. Importantly, there were only five cases of pancreatic cancer in this study; however, considering the overall large denominator of AIP patients at risk, limited follow-up and lack of a control population, it is difficult to understand the true clinical significance of this finding. Additional studies with longer follow-up will help refine our understanding of these long-term sequelae.

We estimate that this multinational collaboration of many of the academic foci for AIP permitted analysis of a significant proportion of the world's current AIP population. Our utilisation of the recently developed ICDC permitted study of patients with a unified set of diagnostic criteria. Our data must be interpreted with caution recognising this collaboration could inadvertently introduce heterogeneity in disease on the basis of unknown ethnic differences in the natural history of disease, as well as variations in the standards of care regarding disease evaluation and follow-up.

Although the basic clinical profiles and initial treatment strategies for AIP are generally understood, many questions remain. The importance of steroid treatment at disease onset is commonly accepted, however whether or not one could use a lower steroid dose has not been systematically evaluated and may potentially decrease treatment-related morbidity. The prediction of disease relapse remains inadequate. Except for IgG4-related SC, no clinical factor has been consistently demonstrated to predict subsequent relapse. Finally, it remains unclear whether or not maintenance therapy (using either low-dose steroids or an immunomodulator) actually prolongs relapse-free survival, thereby altering the course of disease.

In summary, in this multinational analysis of more than 1000 patients with AIP we have shown that most patients are treated with steroids for predominantly pancreatobiliary manifestations of their disease. Initial and subsequent treatment responses to steroid therapy are exceedingly high, so the diagnosis should be reconsidered if patients do not respond to steroids. Relapses occur in a substantial proportion of patients and typically involve the pancreas and/or biliary system. Pancreatic duct stones and malignancies are two potential long-term sequelae, which require ongoing surveillance to further understand their full clinical significance. We are hopeful that multinational collaborations, such as the present one, will provide opportunities to better understand this disease, and permit a long-awaited randomised treatment trial.

## Supplementary Material

Web supplement

## Supplementary Material

Web supplement

## Supplementary Material

Web supplement

## Supplementary Material

Web supplement
