# Supplementary material for: Long-term outcomes of autoimmune pancreatitis: a multicentre, international analysis
Source: Gut. 2012 Dec 11;62(12):1771–6. doi: 10.1136/gutjnl-2012-303617 (PMC3862979; doi:10.1136/gutjnl-2012-303617)
Supplement: Web supplement [file gutjnl-2012-303617-s1.pdf]

Diagnosis	Primary Basis for Diagnosis	Imaging Evidence	Collateral Evidence
Definitive type 1 AIP	Histology	Typical/indeterminate	Histologically confirmed LPSP (level 1 H)
	Imaging	Typical<br>Indeterminate	Any non-D level 1/level 2<br>Two or more from level 1 (+level 2 D*)
	Response to steroid	Indeterminate	Level 1 S/OOI + Rt or level 1 D + level 2 S/OOI/H + Rt
Probable type 1 AIP		Indeterminate	Level 2 S/OOI/H + Rt

*Level 2 D is counted as level 1 in this setting.
