# Supplementary material for: Long-term outcomes of autoimmune pancreatitis: a multicentre, international analysis
Source: Gut. 2012 Dec 11;62(12):1771–6. doi: 10.1136/gutjnl-2012-303617 (PMC3862979; doi:10.1136/gutjnl-2012-303617)
Supplement: Web supplement [file gutjnl-2012-303617-s2.pdf]

Criterion		Level 1	Level 2
P	Parenchymal imaging	Typical:<br>Diffuse enlargement with delayed enhancement (sometimes associated with rim-like enhancement)	Indeterminate (including atypical[†]):<br>Segmental/focal enlargement with delayed enhancement
D	Ductal imaging (ERP)	Long (>1/3 length of the main pancreatic duct) or multiple strictures without marked upstream dilatation	Segmental/focal narrowing without marked upstream dilatation (duct size, <5 mm)
S	Serology	IgG4, >2× upper limit of normal value	IgG4, 1–2× upper limit of normal value
OOI	Other organ involvement	a or b<br>a. Histology of extrapancreatic organs<br>Any three of the following:<br>(1) Marked lymphoplasmacytic infiltration with fibrosis and without granulocytic infiltration<br>(2) Storiform fibrosis<br>(3) Obliterative phlebitis<br>(4) Abundant (>10 cells/HPF) IgG4-positive cells<br>b. Typical radiological evidence<br>At least one of the following:<br>(1) Segmental/multiple proximal (hilar/intrahepatic) or proximal and distal bile duct stricture<br>(2) Retroperitoneal fibrosis	a or b<br>a. Histology of extrapancreatic organs including endoscopic biopsies of bile duct[‡]:<br>Both of the following:<br>(1) Marked lymphoplasmacytic infiltration without granulocytic infiltration<br>(2) Abundant (>10 cells/HPF) IgG4-positive cells<br>b. Physical or radiological evidence<br>At least one of the following:<br>(1) Symmetrically enlarged salivary/lachrymal glands<br>(2) Radiological evidence of renal involvement described in association with AIP
H	Histology of the pancreas	LPSP (core biopsy/resection)<br>At least 3 of the following:<br>(1) Periductal lymphoplasmacytic infiltrate without granulocytic infiltration<br>(2) Obliterative phlebitis<br>(3) Storiform fibrosis<br>(4) Abundant (>10 cells/HPF) IgG4-positive cells	LPSP (core biopsy)<br>Any 2 of the following:<br>(1) Periductal lymphoplasmacytic infiltrate without granulocytic infiltration<br>(2) Obliterative phlebitis<br>(3) Storiform fibrosis<br>(4) Abundant (>10 cells/HPF) IgG4-positive cells
		Diagnostic steroid trial	
Response to steroid (Rt)*		Rapid (≤2 wk) radiologically demonstrable resolution or marked improvement in pancreatic/extrapancreatic manifestations	

*Diagnostic steroid trial should be conducted carefully by pancreatologists with caveats (see text) only after negative workup for cancer including endoscopic ultrasound-guided fine needle aspiration.

[†]Atypical: Some AIP cases may show low-density mass, pancreatic ductal dilatation, or distal atrophy. Such atypical imaging findings in patients with obstructive jaundice and/or pancreatic mass are highly suggestive of pancreatic cancer. Such patients should be managed as pancreatic cancer unless there is strong collateral evidence for AIP, and a thorough workup for cancer is negative (see algorithm).

[‡]Endoscopic biopsy of duodenal papilla is a useful adjunctive method because ampulla often is involved pathologically in AIP.
