# Supplementary material for: Long-term outcomes of autoimmune pancreatitis: a multicentre, international analysis
Source: Gut. 2012 Dec 11;62(12):1771–6. doi: 10.1136/gutjnl-2012-303617 (PMC3862979; doi:10.1136/gutjnl-2012-303617)
Supplement: Web supplement [file gutjnl-2012-303617-s3.pdf]

Diagnosis	Imaging Evidence	Collateral Evidence
Definitive type 2 AIP	Typical/indeterminate	Histologically confirmed IDCP (level 1 H) or clinical inflammatory bowel disease + level 2 H + Rt
Probable type 2 AIP	Typical/indeterminate	Level 2 H/clinical inflammatory bowel disease + Rt
