# Supplementary material for: Long-term outcomes of autoimmune pancreatitis: a multicentre, international analysis
Source: Gut. 2012 Dec 11;62(12):1771–6. doi: 10.1136/gutjnl-2012-303617 (PMC3862979; doi:10.1136/gutjnl-2012-303617)
Supplement: Web supplement [file gutjnl-2012-303617-s4.pdf]

	Criterion	Level 1	Level 2
P	Parenchymal imaging	Typical:<br>Diffuse enlargement with delayed enhancement (sometimes associated with rim-like enhancement)	Indeterminate (including atypical†):<br>Segmental/focal enlargement with delayed enhancement
D	Ductal imaging (ERP)	Long (>1/3 length of the main pancreatic duct) or multiple strictures without marked upstream dilatation	Segmental/focal narrowing without marked upstream dilatation (duct size, <5 mm)
OOI	Other organ involvement		Clinically diagnosed inflammatory bowel disease
H	Histology of the pancreas (core biopsy/resection)	IDCP:<br>Both of the following:<br>(1) Granulocytic infiltration of duct wall (GEL) with or without granulocytic acinar inflammation<br>(2) Absent or scant (0–10 cells/HPF) IgG4-positive cells	Both of the following:<br>(1) Granulocytic and lymphoplasmacytic acinar infiltrate<br>(2) Absent or scant (0–10 cells/HPF) IgG4-positive cells
		Diagnostic steroid trial	
Response to steroid (Rt)*		Rapid (≤2 wk) radiologically demonstrable resolution or marked improvement in manifestations	

*Diagnostic steroid trial should be conducted carefully by pancreatologists with caveats (see text) only after negative workup for cancer including endoscopic ultrasound-guided fine needle aspiration.

†Atypical: Some AIP cases may show low-density mass, pancreatic ductal dilatation, or distal atrophy. Such atypical imaging findings in patients with obstructive jaundice and/or pancreatic mass are highly suggestive of pancreatic cancer. Such patients should be managed as pancreatic cancer unless there is strong collateral evidence for AIP, and a thorough workup for cancer is negative (see algorithm).
